# Supplementary material for: Demonstration of relativistic electron beam focusing by a laser-plasma lens
Source: Nat Commun. 2015 Apr 16;6:6860. doi: 10.1038/ncomms7860 (PMC4410646; doi:10.1038/ncomms7860)
Supplement: Supplementary Information — Supplementary Figure 1 [file ncomms7860-s1.pdf]

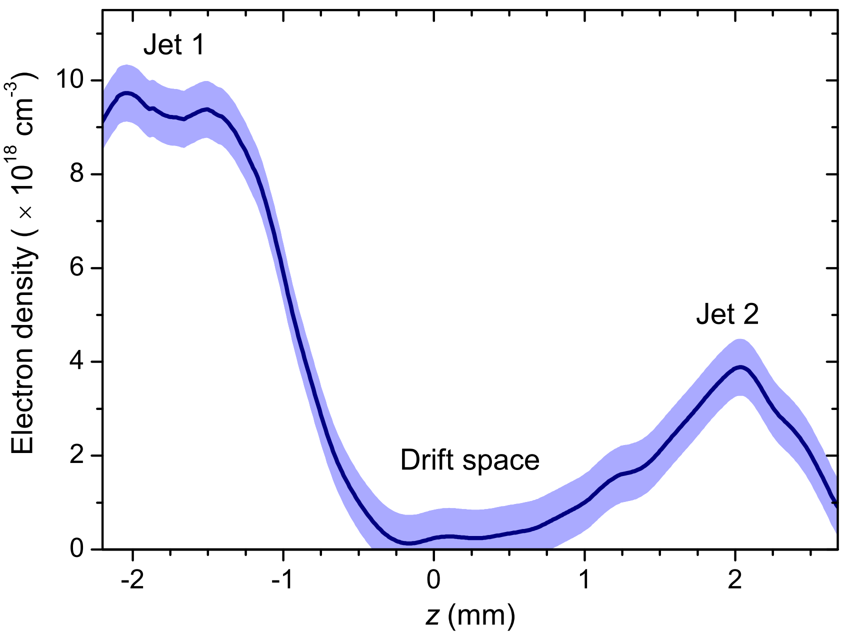

Supplementary Figure 1 | Measured density profile The density profile was measured from interferometry and Abel inversion. In this case, the gap between the two gas jets is $L \approx 2.3$ mm (measured at half maximum). In the experiment the density of the first jet was constant, while the density of the second jet was tuned from 0 up to $6.2 \pm 0.5 \times 10^{18}$ cm^{-3}. The filled area indicates the uncertainty on the measurement.
